# Impact of graft and tunnel orientation on patient-reported outcome in anterior cruciate ligament reconstruction using bone-patellar tendon-bone autografts

**DOI:** 10.1186/s13018-018-0954-3

**Published:** 2018-10-03

**Authors:** Christoph J. Laux, Erika J. Ulbrich, Gustav Andreisek, Magda Marcon, Michael A. Fischer, Tarun Mehra, Bernhard D. Ciritsis

**Affiliations:** 1 Department of Trauma Surgery, University Hospital Zurich, University of Zurich, Zurich, Switzerland; 2 Institute of Diagnostic and Interventional Radiology, University Hospital Zurich, University of Zurich, Zurich, Switzerland; 3 Medical Directorate, University Hospital Zurich, University of Zurich, Zurich, Switzerland

**Keywords:** Anterior cruciate ligament, ACL reconstruction, Bone-patellar tendon-bone, Graft orientation, Outcome

## Abstract

**Background:**

The optimal positioning of anterior cruciate ligament graft is still controversially discussed. We therefore wanted to determine the tunnel-to-joint (TJA), tunnel-to-shaft (TSA), and graft-tunnel divergence angles which would provide the best outcome, determined by the KOOS (Knee Injury and Osteoarthritis Outcome Score). This study evaluated the clinical influence of graft orientation as measured with the KOOS questionnaire in patients with anterior cruciate ligament reconstruction with bone-patellar tendon-bone autografts.

**Methods:**

We designed a prospective cohort study, with a 1 ¼ year recruitment phase from March 2011 to July 2012 and a minimal follow-up period of 1 year. Inclusion criteria were patients ≥ 18 years of age receiving an ACL reconstruction with bone-patellar tendon-bone autografts at our institution after having suffered an acute ACL rupture. The primary outcome was the KOOS. Independent variables were patient age, gender, laterality of rupture, mechanism of trauma, and type of femoral and tibial fixation, as well as sagittal graft-tunnel divergence, TJA, and TSA, the latter two being assessed on coronal slices of magnetic resonance imaging. Equations modeling the relationship between TJA, TSA, and graft-tunnel divergence with the KOOS overall score were fitted, and the optimum angles were mathematically determined.

**Results:**

In total, 31 patients were included in our study. Our cohort with a median age of 28 years was predominantly male. The mathematically determined optimal placement of the implant in the coronal plane was a TJA of 74.8°, a TSA of 80.1°, and a graft-tunnel divergence angle of 8.5°.

**Conclusion:**

With regard to patient-reported outcome, the optimal graft orientation is provided by a coronal tunnel-to-shaft angle of 80° and tunnel-to-joint angle of 75°, respectively. Interestingly, in our series, patients reported best clinical outcomes with a sagittal graft-tunnel divergence. These results should be validated in larger studies.

**Electronic supplementary material:**

The online version of this article (10.1186/s13018-018-0954-3) contains supplementary material, which is available to authorized users.

## Background

Anterior cruciate ligament (ACL) rupture is a frequent sports injury that often leads to post-traumatic knee instability and secondary knee damage with meniscal tears and articular cartilage injuries [1]. The primary goal of ACL reconstruction is to restore knee biomechanics, ensure full functionality permitting the complete resumption of physical activities, and maximize health-related quality of life [2]. Thus, ACL reconstruction has evolved to be a common procedure in orthopedic surgery. Currently, when aiming for an autologous reconstruction, either bone-patellar tendon-bone (BPTB) or hamstring autografts are deployed. The biomechanical properties of both BPTB and hamstring autograft have been investigated to comply well with the native ACL in terms of ultimate failure strength and mean stiffness [3]. Despite increasing knowledge on knee biomechanics and the ACL architecture as well as widespread practice, many aspects regarding the operative technique of ACL reconstruction remain controversial. This also includes the femoral insertion point and the resulting graft orientation. Based on biomechanical studies, a more oblique graft orientation is considered to better restore rotational stability and prevent the pivot-shift phenomenon when compared to vertical graft placements in the femoral notch [4, 5].

The self-administered easy to use Knee Injury and Osteoarthritis Outcome Score (KOOS) developed in 1998 assesses all of the aforementioned outcomes and can be used for assessment of ACL reconstruction outcome [6]. The KOOS has been shown to be a valid, reliable, and responsive outcome measure in numerous studies [7–10].

The objective of this study therefore was to determine the optimal surgical implantation technique with regard to surgical outcome 1 year after surgery, determined by the implant or tunnel angles associated with the minimum KOOS overall score.

## Methods

### Study design

We designed a prospective cohort study, with a 1 ¼ year recruitment phase from March 2011 to July 2012 and a minimal follow-up period of 1 year. Inclusion criteria were patients ≥ 18 years of age receiving an ACL reconstruction with bone-patellar tendon-bone autografts at our institution after having suffered an acute ACL rupture. The primary outcome was the KOOS after a minimum follow-up period of 1 year, after which patients were requested to complete the KOOS questionnaire. MR imaging was obtained on the day of filling in the KOOS questionnaire. Exclusion criteria were prior ACL reconstruction (*n* = 5), additional rheumatic/musculoskeletal disorders (*n* = 3), and inadequate image quality in any sequence (sagittal T2-weighted fat saturated, coronal and sagittal proton density, and axial fat saturated proton density sequences) of magnetic resonance imaging (MRI) (*n* = 6). MR imaging was performed using a 1.5 Tesla MR unit (Signa Echospeed EXCITE HDxt; GE Healthcare, Waukesha, Wisconsin, USA). Patients who did not complete the 1-year follow-up period (*n* = 12) or withdrew their consent to participate in the study (*n* = 3) were also excluded from the study, as were patients who did not complete the KOOS (*n* = 2). One patient, who has sustained a re-rupture within the follow-up period, was excluded due to missing assessability of the graft orientation. In total, 63 patients receiving ACL reconstruction at our center during the aforementioned timeframe were screened, of which 31 were included in the final analysis. Both clinicians and patients were blinded as to the results of the MRI assessments to avoid bias.

### Variables

**Fig. 1:**
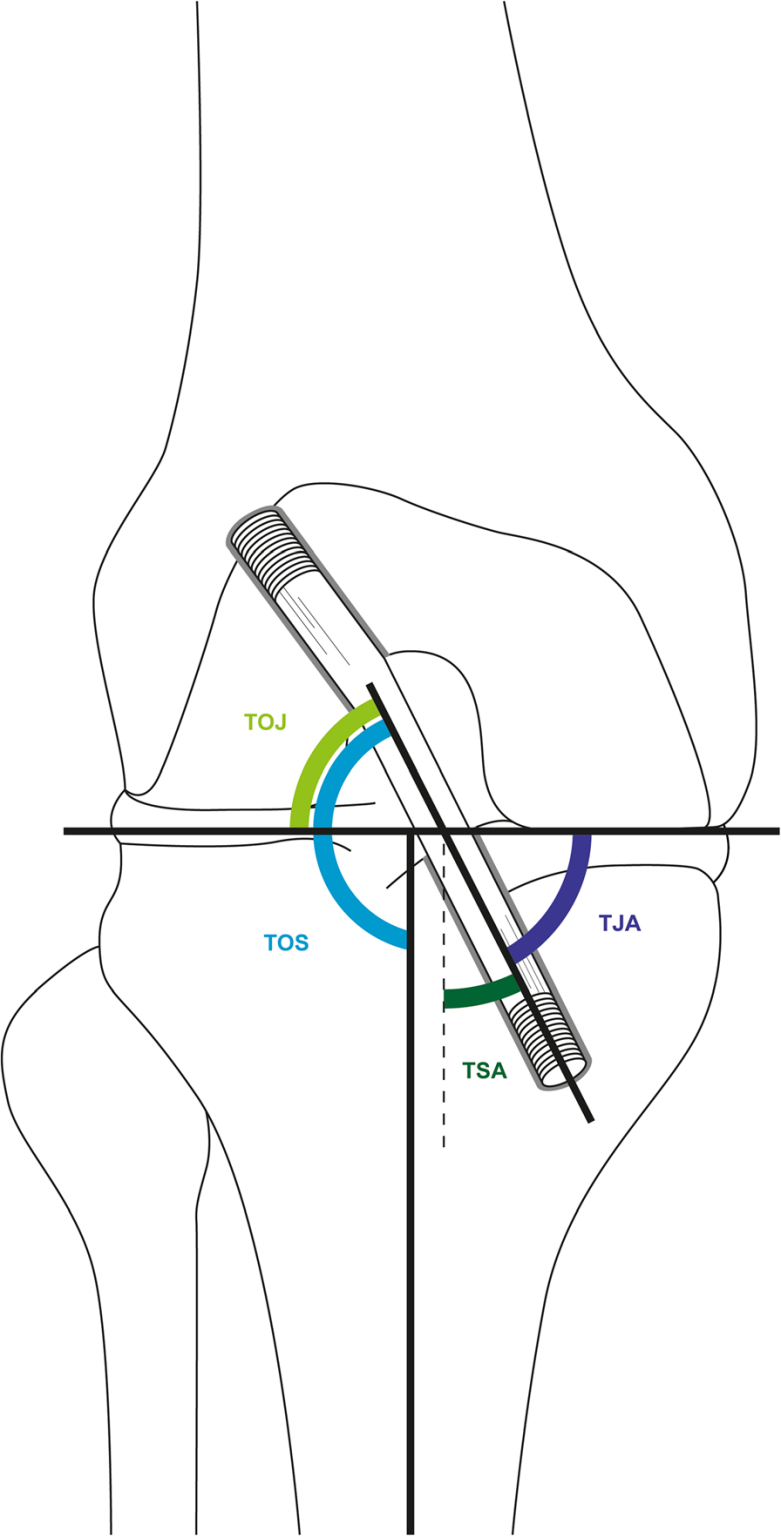
Illustration of ascertained coronal angles: transplant orientation to joint (TOJ), transplant orientation to shaft (TOS), tunnel-to-joint angle (TJA), and tunnel-to-shaft angle (TSA)

**Fig. 2:**
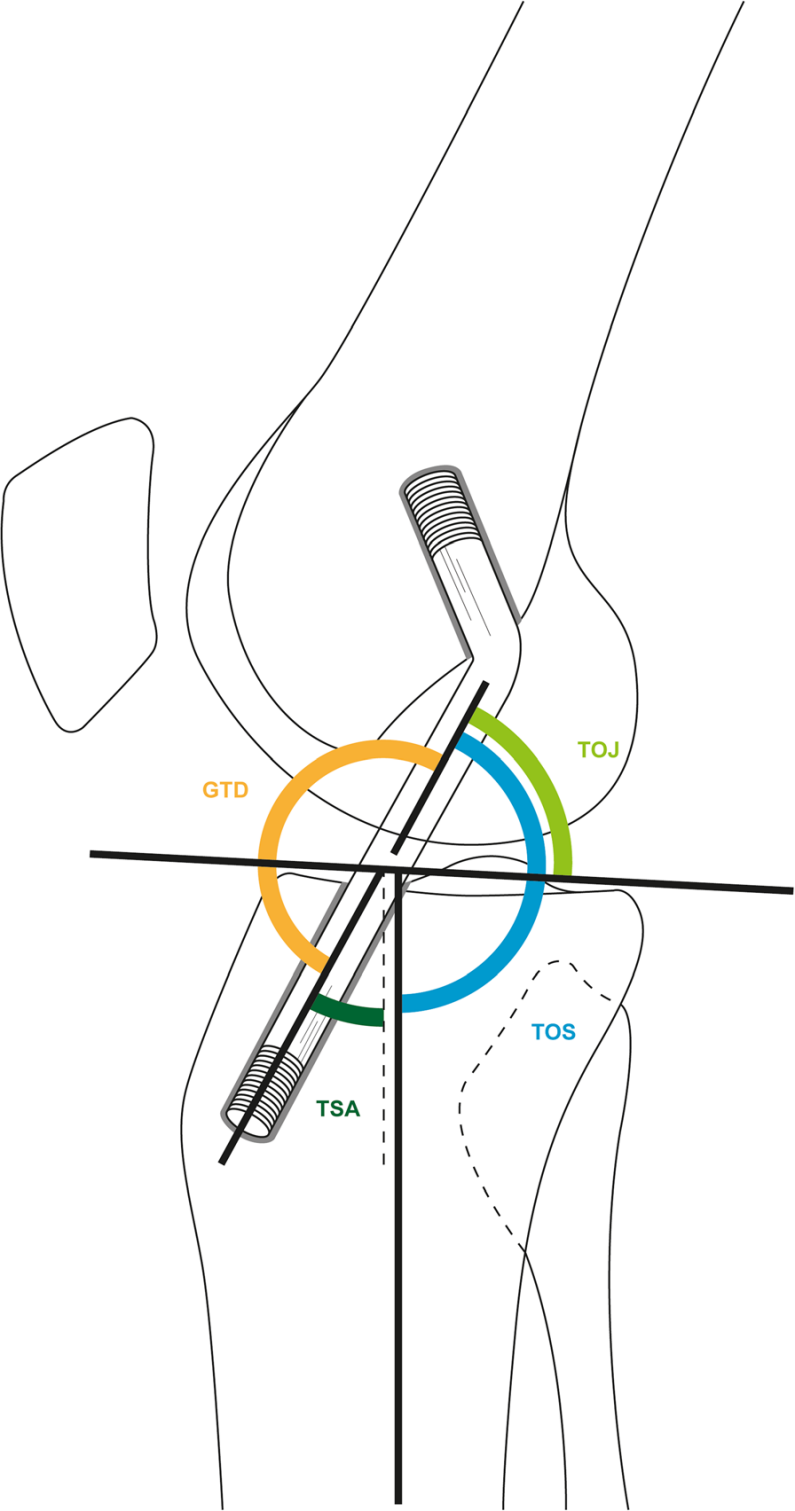
Illustration of ascertained sagittal angles: transplant orientation to joint (TOJ), transplant orientation to shaft (TOS), tunnel-to-shaft angle (TSA), and graft kinging angle (GTD)

Primary outcome was the KOOS overall score determined by self-assessment approximately 1 year after surgery (mean follow-up 19 ± 3.7 months). The patient-administered KOOS questionnaire consists of five subscales (pain, other symptoms, function in daily life, function in sports and recreation, and knee-related quality of life). Patient age, gender, laterality of rupture, mechanism of trauma, type of femoral and tibial fixation, transplant orientation to joint (TOJ), transplant orientation to shaft (TOS), tunnel-to-joint (TJA), and tunnel-to-shaft (TSA) angles as well as sagittal graft-tunnel divergence (all assessed in MRI images taken post-surgically) were included in our analysis. The angles defined are illustrated in Figs. 1 and 2, respectively. In addition, surgical reports were reviewed to collect data on concomitant injuries such as meniscal tears or cartilage lesions. Measurements of ACL graft orientation were performed by a skilled musculoskeletal radiologist with 14 years of professional experience on sagittal and coronal proton density weighted sequences based on the method previously described by Scanlan et al. [11]. However, since the sagittal joint line at the tunnel exit is difficult to determine in tomographic images, we also ascertained the tunnel and graft orientation with reference to the tibial shaft. Graft-tunnel divergence was considered being the sagittal angulation of the graft when exiting the tibial tunnel and thus was calculated as the difference between tunnel orientation and graft orientation (with reference to the tibial shaft) in the sagittal slices.

### Statistical analysis

Data was collected in spreadsheets (Microsoft Excel 2010, Microsoft Corporation, Redmond, USA, version 14.0.7140.5002). All statistical analyses were performed using IBM SPSS Statistics version 22 (IBM Corporation, Armonk, USA, Release 22.0.0.1). Distribution of independent variables was assessed with descriptive statistics. The relationship between TSA, TJA, graft-tunnel divergence angle, and the primary outcome, the KOOS, was graphically assessed. Presuming there is an optimum for which the KOOS would be the lowest (in absolute numbers, not in a normalized scale), we fitted quadratic functions of the type *y* = *k* + *ß*_1_ × *x* + *ß*_2_ × *x*^2^ to model the relationship between the KOOS and TSA or TJA respectively. We fitted linear and quadratic functions to model the relationship between the KOOS and graft-tunnel divergence angle. The optimal TJA, TSA, and graft-tunnel divergence angles in the coronal plane were determined mathematically by resolving the curve’s equation for the minimum KOOS, i.e., where the derivative of the independent variable was 0.

## Results

In total, 31 patients were included in our analysis. Our cohort comprised younger, mostly male patients with a normal body mass index. The median age at injury was 28 years. All but two ACL ruptures were sports-related, the most frequent being soccer (36%) followed by alpine skiing (26%). Concomitant meniscal tears were frequently diagnosed (48%), whereas cartilage lesions (maximum grade II according to Outerbridge) were only found in two patients (6%). However, the KOOS overall score was not significantly different between patients with and without concomitant injuries. Femoral fixation was performed with screws of 6–7 mm in width and 19 or 23 mm in length and tibial fixation with screws 7–9 mm in width and 23 mm in length. An ACL implant with a thickness of 9 mm was inserted in 24 patients (77%), and an ACL implant of 8 mm thickness was used in 4 patients (13%). Further ACL implant thicknesses employed were 8.5 mm (2 patients) and 7.0 mm (1 patient). Patient characteristics are summarized in Table 1. The complete list of patients can be found in Additional file 1: Table S1.

**Table 1:** Patient characteristics (*n* = 31)

Age (years)	28 (24–32)
Female gender	12 (39%)
BMI	24.2 (22.7–25.9)
Mean follow-up	19 months (± 3.7)
TOJ (°)	
Coronal plane	77.9 (73.7–81.4)
Sagittal plane	59.7 (57.1–62.9)
TOS (°)	
Coronal plane	81.5 (76.7–84.1)
Sagittal plane	57.1 (53.8–60.7)
TJA (°)	
Coronal plane	73.4 (70.0–79.5)
TSA (°)	
Coronal plane	74.9 (72.8–81.5)
Sagittal plane	61.6 (58.765.5)
Graft-tunnel divergence (°)	6.1 (3.6–13.0)
KOOS	46.0 (42.0–55.0)

Results are depicted in absolute numbers (% of total) or median (interquartile range). KOOS is not normalized

*TJA* tunnel-to-joint angle, *TSA* tunnel-to-shaft angle

**Fig. 3:**
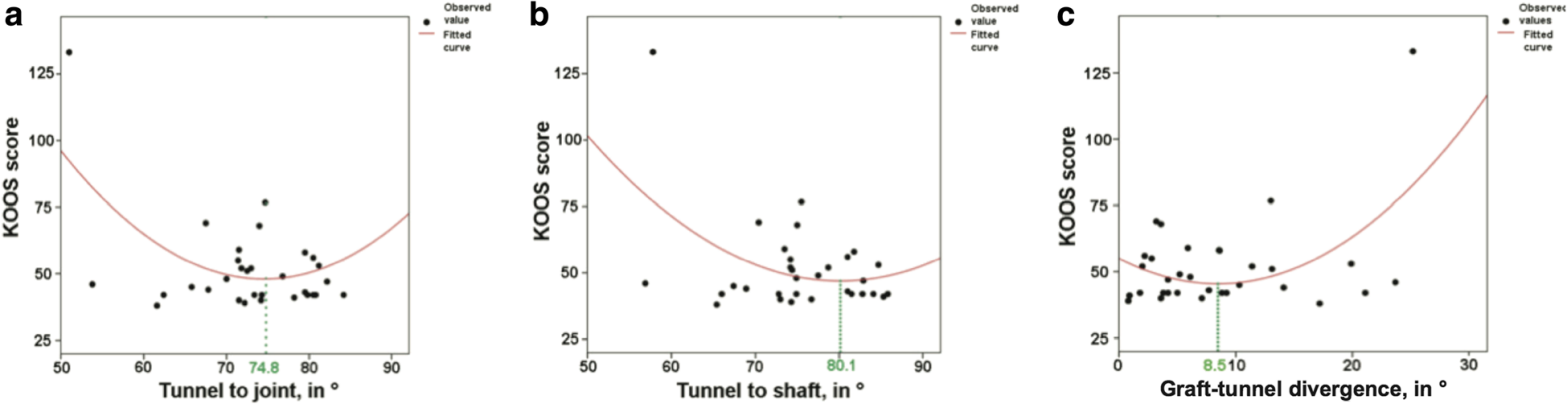
Curve fits for KOOS in function of coronal **a** tunnel-to-joint angle (TJA) and **b** tunnel-to-shaft angle (TSA) and **c** sagittal graft-tunnel divergence (GTD). All three estimated equations are quadratic. The mathematically determined optimum TSA, TJA, and graft kinking values for achieving the best post-surgical results (lowest KOOS, no normalized scale) are marked in green

We modeled the relationship between the KOOS overall score (dependent variable) and graft-tunnel divergence (independent variable) with quadratic and linear equations. As the *R* squared was higher for the quadratic model (0.25 vs. 0.13, *p* = 0.02 vs. *p* = 0.04), we chose the quadratic equation to model the relationship between the KOOS and graft-tunnel divergence. We also modeled the relationship between the KOOS (dependent variable) and TSA as well as TJA (on coronal planes respectively) with quadratic equations. The quadratic equation of coronal TOJ and TOS angles showed an insufficient curve fit (*R* squared 0.012 and 0.023, respectively) and no significant *p* values. Thus, the derivation of optimum values using the parameter estimates was not meaningful for TOJ and TOS angles. The summaries of the curve estimations are depicted in Table 2. The corresponding curves are plotted in Fig. 3.

**Table 2:** Summary of the model equations, the KOOS overall score being the dependent variable (*n* = 31)

	Model summary					Parameter estimates		
	*R* squared	*F* statistic	DoF (*ß*_1_)	DoF (*ß*_2_)	*p* value	Constant (*k*)	*ß*_1_	*ß*_2_
TJA	.315	6.438	2	28	.005	492.508	− 11.926	.080
TSA	.196	3.406	2	28	.047	434.071	− 9.663	.060
TOJ	.012	0.171	2	28	.843	82.087	− .151	.001
TOS	.023	0.328	2	28	.723	87.172	− .223	.001
Graft-tunnel divergence	.250	4.675	2	28	.018	55.012	− 2.259	.133

All equations are of the type *y* = *k* + *ß*_1_ × *x* + *ß*_2_ × *x*^2^, where *y* is the dependent variable, *x* is the independent variable; *ß* denotes a coefficient, and *k* is a constant

*DoF* degrees of freedom

With the optimum outcome being the minimum KOOS, the optimal implant angles can be mathematically determined by resolving the equation for *y*’ = *f*’(*x*) = 0. In the coronal plane, the optimal angles hence were a TJA of 74.8° and a TSA of 80.1°. In the sagittal plane, the curve morphology did not admit the abovementioned calculations of optimum values due to its convexity. The optimal graft-tunnel divergence angle was calculated to be 8.5°.

## Discussion

Assessing the relationship between post-surgical outcome determined by the KOOS and surgical ACL implantation technique determined by the TSA, TJA, and graft-tunnel divergence angles in a prospective cohort study with 31 patients, we mathematically determined the optimum angles to be 80.1°, 74.8°, and 8.5°, respectively.

Although calculated in a small study population, the sagittal graft-tunnel divergence angle was conspicuous. Derived from the surgical technique of implantation with drilling a bone tunnel over a leading wire, we expected the optimal graft-tunnel divergence angle to lean towards zero. Especially when considering the supine position of the patient during the image acquisition, a posterior graft-tunnel divergence seems surprising. Graft-tunnel divergence at the tibial site could possibly be associated to tibial tunnel widening through a wiper effect. Eventually, this observation and its biomechanical or clinical relevance need to be verified and further investigated in larger studies.

This study certainly has limitations. The small sample size substantially limited the statistical possibilities and the validity of the results. However, as this is a primarily radiological investigation, we did not want to compromise the imaging quality and excluded patients with poor post-surgical imaging. Therefore, we accepted a high dropout rate. The recruitment process is illustrated below. Despite the small sample size and weak statistics, our results did not show significant differences when compared to other series [11].

The study design also contains the weakness of acquiring the imaging at different points in the course of treatment. The postoperative MRI was obtained between 11 and 23 months (median 20 months) postoperatively. Even though it is doubtful, that the graft orientation changes during this time, imaging earlier in the postoperative course might have allowed inclusion of patients that have sustained a graft re-rupture (*n* = 1). This might have yielded valuable information on the desirable graft orientation.

Even though the exact time required for ligament remodeling is not known and most likely highly individual, we considered a least follow-up period of 1 year to be sufficient. Radiologically, an incorporation of the remnant stump is seen after 8 months [12]. As stated above, the confounding effect of implant remodeling on the graft orientation is questionable. In our collective, only 12% of patients did not return to sports at this time (2 soccer players, 1 skier, 1 motorcyclist).

For our analyses, we did measure the intraarticular graft orientation as proposed by Scanlan et al. [11]. This method uses the coronal and sagittal joint line in three-dimensional (3D) MR models as respective reference. However, 3D models were not available and the overall sagittal joint line is difficult to assess at a single slice. We did address this problem by measuring the graft orientation with reference to the tibial shaft, which is easier to realize and can also be applied in sectional images. For this reason, the sagittal graft-tunnel divergence is calculated with reference to the tibial shaft.

## Conclusion

We clinically and radiologically analyzed 31 patients with a minimum follow-up of 1 year after ACL reconstruction using a bone-patellar tendon-bone autograft. With regard to patient-reported outcome, the optimal graft orientation is provided by a coronal tunnel-to-shaft angle of 80° and tunnel-to-joint angle of 75°, respectively. Interestingly, in our series, patients reported best clinical outcomes with a sagittal graft-tunnel divergence of 8.5°. These results should be validated in larger studies.

## Additional file

Additional file 1: **Table S1.** Patient cohort (*n* = 31). (DOC 111 kb)

## References

[1] Zantop T, Petersen W, Sekiya JK, Musahl V, Fu FH (2006). Anterior cruciate ligament anatomy and function relating to anatomical reconstruction. Knee Surg Sports Traumatol Arthrosc.

[2] Goebel J. C., Bolbos R., Pham M., Galois L., Rengle A., Loeuille D., Netter P., Gillet P., Beuf O., Watrin-Pinzano A. (2010). In vivo high-resolution MRI (7T) of femoro-tibial cartilage changes in the rat anterior cruciate ligament transection model of osteoarthritis: a cross-sectional study. Rheumatology.

[3] Wilson Tad W., Zafuta Michael P., Zobitz Mark (1999). A Biomechanical Analysis of Matched Bone-Patellar Tendon-Bone and Double-Looped Semitendinosus and Gracilis Tendon Grafts. The American Journal of Sports Medicine.

[4] Jepsen CF, Lundberg-Jensen AK, Faunoe P (2007). Does the position of the femoral tunnel affect the laxity or clinical outcome of the anterior cruciate ligament-reconstructed knee? A clinical, prospective, randomized, double-blind study. Arthroscopy.

[5] Lee MC, Seong SC, Lee S, Chang CB, Park YK, Jo H, Kim CH (2007). Vertical femoral tunnel placement results in rotational knee laxity after anterior cruciate ligament reconstruction. Arthroscopy.

[6] Roos EM, Roos HP, Lohmander LS, Ekdahl C, Beynnon BD (1998). Knee Injury and Osteoarthritis Outcome Score (KOOS)—development of a self-administered outcome measure. J Orthop Sports Phys Ther.

[7] Roos EM, Toksvig-Larsen S (2003). Knee injury and Osteoarthritis Outcome Score (KOOS) - validation and comparison to the WOMAC in total knee replacement. Health Qual Life Outcomes.

[8] Salavati M, Akhbari B, Mohammadi F, Mazaheri M, Khorrami M (2011). Knee injury and Osteoarthritis Outcome Score (KOOS); reliability and validity in competitive athletes after anterior cruciate ligament reconstruction. Osteoarthr Cartil.

[9] White DK, Master H (2016). Patient-reported measures of physical function in knee osteoarthritis. Rheum Dis Clin N Am.

[10] Collins NJ, Prinsen CAC, Christensen R, Bartels EM, Terwee CB, Roos EM (2016). Knee Injury and Osteoarthritis Outcome Score (KOOS): systematic review and meta-analysis of measurement properties. Osteoarthr Cartil.

[11] Scanlan SF, Blazek K, Chaudhari AMW, Safran MR, Andriacchi TP (2009). Graft orientation influences the knee flexion moment during walking in patients with anterior cruciate ligament reconstruction. Am J Sports Med.

[12] Whitehead TS (2013). Failure of anterior cruciate ligament reconstruction. Clin Sports Med.

