# Supplementary material for: Impact of graft and tunnel orientation on patient-reported outcome in anterior cruciate ligament reconstruction using bone-patellar tendon-bone autografts
Source: J Orthop Surg Res. 2018 Oct 3;13:245. doi: 10.1186/s13018-018-0954-3 (PMC6171132; doi:10.1186/s13018-018-0954-3)
Supplement: Supplementary file 1 — Table S1. Patient cohort (n = 31). (DOC 111 kb) [file 13018_2018_954_MOESM1_ESM.doc]

**Table S1** Patient cohort (n=31)

| **#** | **Sex** | **Height (cm)** | **Weight (kg)** | **BMI (kg/m^2^)** | **Age (years)** | **Laterality of ACL rupture** | **Trauma Mechanism** | **Time to treatment (days)** | **Follow-up (months)** | **Implant thickness (mm)** | **Concomitant injuries** | **TOJ (°) COR** | **TOJ (°) SAG** | **TOS (°) COR** | **TOS (°) SAG** | **TJA (°) COR** | **TSA (°) COR** | **TSA (°)**  **SAG** | **GTD SAG** | **KOOS overall score** |
| --- | --- | --- | --- | --- | --- | --- | --- | --- | --- | --- | --- | --- | --- | --- | --- | --- | --- | --- | --- | --- |
| 1 | m | 190 | 94 | 26.0 | 28 | L | Soccer | 7 | 23 | 9 | - | 79.9 | 56.9 | 82.3 | 60.7 | 79.8 | 84.1 | 54.1 | 1.8 | 43 |
| 2 | f | 161 | 68 | 26.2 | 24 | L | Skiing | 39 | 23 | 8 | - | 82.7 | 57.1 | 88.7 | 61.5 | 79.5 | 81.0 | 61.7 | 7.7 | 43 |
| 3 | m | 166 | 60 | 21.8 | 29 | R | Soccer | 71 | 23 | 9 | LM | 87.1 | 57.8 | 88.6 | 59.6 | 74.7 | 75.6 | 50.6 | 13.0 | 77 |
| 4 | m | 172 | 83 | 28.1 | 28 | R | Soccer | 180 | 23 | 9 | MM, central MF I° | 73.7 | 57.5 | 76.7 | 53.8 | 73.1 | 78.7 | 69.3 | 2.0 | 52 |
| 5 | m | 181 | 75 | 22.9 | 36 | L | Hockey | 55 | 23 | 9 | - | 67.5 | 46.6 | 69.6 | 41.8 | 74.2 | 76.7 | 70.8 | 7.1 | 40 |
| 6 | m | 168 | 66 | 23.4 | 28 | R | Basketball | 13 | 23 | 9 | MM, LM | 81.4 | 59.7 | 82.6 | 59.7 | 61.6 | 65.4 | 66.6 | 17.2 | 38 |
| 7 | m | 180 | 86 | 26.5 | 31 | R | Sport - unspecified | 354 | 23 | 8 | MM | 77.2 | 62.9 | 79.5 | 55.9 | 71.5 | 73.6 | 58.6 | 5.9 | 59 |
| 8 | m | 162 | 66 | 25.1 | 49 | R | Skiing | 199 | 20 | 9 | - | 82.6 | 69.9 | 82.8 | 71.8 | 76.8 | 77.6 | 70.2 | 5.2 | 49 |
| 9 | f | 163 | 64 | 24.1 | 31 | R | Skiing | 33 | 22 | 9 | LM | 81.4 | 56.4 | 84.1 | 57.3 | 74.3 | 74.9 | 63.3 | 9.2 | 42 |
| 10 | m | 176 | 77 | 24.9 | 34 | L | Skiing | 123 | 22 | 9 | MM | 79.0 | 60.2 | 81.6 | 61.3 | 73.4 | 72.8 | 63.2 | 8.8 | 42 |
| 11 | m | 170 | 72 | 24.9 | 25 | R | Household | 12 | 23 | 9 | - | 82.7 | 61.3 | 87.5 | 65.9 | 72.5 | 74.4 | 68.0 | 13.1 | 51 |
| 12 | f | 156 | 63 | 25.9 | 22 | L | Soccer | 131 | 23 | 9 | LM | 75.8 | 68.5 | 78.7 | 68.0 | 82.2 | 82.9 | 61.2 | 4.2 | 47 |
| 13 | m | 177 | 77 | 24.6 | 19 | R | Military | 55 | 23 | 8.5 | - | 77.0 | 65.7 | 76.7 | 57.1 | 71.5 | 73.1 | 64.3 | 3.6 | 40 |
| 14 | m | 180 | 80 | 24.7 | 30 | R | Soccer | 96 | 17 | 9 | LM | 77.9 | 59.4 | 84.4 | 53.7 | 78.2 | 85.3 | 48.1 | 0.9 | 41 |
| 15 | f | 169 | 79 | 27.7 | 22 | L | Sport - unspecified | 54 | 15 | 9 | - | 76.2 | 52.7 | 77.8 | 45.6 | 80.8 | 81.6 | 60.7 | 3.8 | 42 |
| 16 | f | 165 | 73 | 26.8 | 36 | R | Soccer | 131 | 21 | 8.5 | - | 79.2 | 59.6 | 83.0 | 55.0 | 51.0 | 57.8 | 65.2 | 25.2 | 133 |
| 17 | f | 165 | 60 | 22.0 | 24 | L | Sport | 51 | 17 | 8 | MM | 78.6 | 58.1 | 83.0 | 51.3 | 67.8 | 68.9 | 60.0 | 14.1 | 44 |
| 18 | f | 163 | 67 | 25.2 | 17 | L | Sport | 2831 | 20 | 9 | - | 86.2 | 61.7 | 87.8 | 57.0 | 80.6 | 82.8 | 61.6 | 5.0 | 42 |
| 19 | m | 195 | 80 | 21.0 | 22 | R | Soccer | 154 | 12 | 9 | - | 77.0 | 62.4 | 81.6 | 59.6 | 84.2 | 85.8 | 60.0 | 4.2 | 42 |
| 20 | f | 169 | 59 | 20.7 | 50 | R | Hiking | 58 | 14 | 9 | MM | 80.7 | 58.6 | 83.2 | 56.3 | 80.5 | 81.0 | 58.7 | 2.2 | 56 |
| 21 | m | 183 | 80 | 23.9 | 21 | L | Handball | 237 | 16 | 9 | MM | 72.8 | 66.0 | 77.0 | 54.7 | 71.4 | 74.2 | 63.3 | 2.8 | 55 |
| 22 | m | 180 | 76 | 23.5 | 30 | R | Soccer | 127 | 12 | 9 | - | 66.1 | 60.6 | 68.8 | 58.7 | 70.1 | 74.9 | 70.3 | 6.1 | 48 |
| 23 | f | 157 | 49 | 19.9 | 24 | R | Soccer | 7 | 13 | 9 | LM | 62.3 | 60.4 | 64.8 | 61.5 | 81.2 | 84.7 | 59.2 | 19.9 | 53 |
| 24 | m | 172 | 80 | 27.0 | 32 | R | Sport - unspecified | 13 | 14 | 9 | - | 84.5 | 50.9 | 87.1 | 40.0 | 62.4 | 66.0 | 54.8 | 21.1 | 42 |
| 25 | m | 183 | 83 | 24.8 | 29 | L | Skiing | 9 | 17 | 9 | - | 79.2 | 57.0 | 78.7 | 47.7 | 74.0 | 75.1 | 66.4 | 3.6 | 68 |
| 26 | f | 170 | 60 | 20.8 | 39 | R | Skiing | 8 | 17 | 9 | MM | 66.7 | 54.2 | 73.2 | 46.4 | 79.6 | 81.8 | 63.0 | 8.6 | 58 |
| 27 | m | 181 | 78 | 23.8 | 32 | R | Soccer | 16 | 17 | 9 | - | 76.2 | 65.3 | 80.6 | 55.6 | 53.8 | 56.9 | 61.5 | 23.7 | 46 |
| 28 | m | 177 | 75 | 23.9 | 18 | L | Soccer | 3 | 22 | 9 | - | 73.5 | 63.2 | 75.1 | 62.0 | 72.2 | 74.3 | 55.3 | 0.8 | 39 |
| 29 | m | 180 | 70 | 21.6 | 27 | L | Skiing | 416 | 17 | 7 | LM | 77.0 | 64.9 | 77.7 | 57.2 | 65.8 | 67.4 | 65.6 | 10.3 | 45 |
| 30 | f | 160 | 62 | 24.2 | 28 | R | Climbing | 11 | 18 | 8 | peripheral MT II° | 73.2 | 59.0 | 73.6 | 59.5 | 67.5 | 70.4 | 61.5 | 3.2 | 69 |
| 31 | f | 173 | 68 | 22.7 | 25 | L | Skiing | 174 | 14 | 9 | LM | 83.3 | 61.5 | 85.6 | 56.4 | 71.8 | 74.2 | 55.9 | 11.4 | 52 |

BMI: body mass index; ACL: anterior cruciate ligament; LM: lateral meniscus; MM: medial meniscus; LF: lateral femoral condyle; MF: medial femoral condyle; LT: lateral tibial plateau; MT: medial tibial plateau; TJA: tunnel to joint; TSA: tunnel to shaft; GTD: graft-tunnel divergence; COR: coronal plane; SAG: sagittal plane; m: male; f: female; L: left; R: right; Cartilage lesions are graded according to the Outerbridge classification. KOOS score is not provided in normalized scale.
